# RiPPAS: A Ring-Based Privacy-Preserving Aggregation Scheme in Wireless Sensor Networks

**DOI:** 10.3390/s17020300

**Published:** 2017-02-07

**Authors:** Kejia Zhang, Qilong Han, Zhipeng Cai, Guisheng Yin

**Affiliations:** 1 College of Computer Science & Technology, Harbin Engineering University, Harbin 150001, China; kejiazhang@hrbeu.edu.cn (K.Z.); yinguisheng@hrbeu.edu.cn (G.Y.); 2 Department of Computer Science, Georgia State University, Atlanta, GA 30303, USA; zcai@gsu.edu

**Keywords:** aggregation, data privacy, wireless sensor networks, ring structure

## Abstract

Recently, data privacy in wireless sensor networks (WSNs) has been paid increased attention. The characteristics of WSNs determine that users’ queries are mainly aggregation queries. In this paper, the problem of processing aggregation queries in WSNs with data privacy preservation is investigated. A Ring-based Privacy-Preserving Aggregation Scheme (RiPPAS) is proposed. RiPPAS adopts ring structure to perform aggregation. It uses pseudonym mechanism for anonymous communication and uses homomorphic encryption technique to add noise to the data easily to be disclosed. RiPPAS can handle both sum() queries and min()/max() queries, while the existing privacy-preserving aggregation methods can only deal with sum() queries. For processing sum() queries, compared with the existing methods, RiPPAS has advantages in the aspects of privacy preservation and communication efficiency, which can be proved by theoretical analysis and simulation results. For processing min()/max() queries, RiPPAS provides effective privacy preservation and has low communication overhead.

## 1. Introduction

A Wireless Sensor Network [1,2,3,4] (WSN) comprises a large number of sensor nodes deployed in the monitoring area. Each sensor node is usually a battery-powered tiny device, which is responsible for sampling some attributes like temperature, humidity, pressure, luminous intensity, voice and image from surrounding area. Sensor nodes use simple radio module to communicate with each other. The nodes’ transmitting range is very limited. After being deployed, all the sensor nodes are self-organized as an Ad-Hoc network. Data exchange between two sensor nodes usually needs to relayed by many intermediate nodes. There is a data processing/storge center called sink or base station, which is responsible for receiving users’ queries, distributing queries to the relating sensor nodes, gathering data from the network and return results to users. Sensor nodes are usually deployed where personnel are difficult to reach, and it is impractical to exchange batteries for them, so how to save energy to prolong the life time of each sensor node becomes the main optimizing goal of WSNs. Research results [5,6] show that among all the operations of sensor nodes, wireless communication is the dominating factor for energy consumption. Thus, in-network query processing is necessary for sake of communication efficiency.

In recent years, data privacy of WSNs gets more and more attention [7,8,9,10]. Many reasons make adversaries interested about some nodes’ data in a WSN. For example, in a WSN deployed in the battle filed, our enemies want to intercept the data of reporting enemies’ invasion; Pharmaceutical advertisers want to get the data of people’s physiological indicators collected from the sensors worn by users; To know the location of wildlife, poachers want to access the data of sensor nodes deployed in the wild for wildlife monitoring. Such application scenarios have a strong need for data privacy protection [11].

In most applications of WSNs, users are more interested in the overall situation of the data of the whole network rather than the data of a certain node. Therefore, the queries issued by users are mainly aggregation queries, e.g., query the average of the temperature sampled by all the nodes or query the minimum value of the humidity sampled by the nodes in a given area. Characteristics of WSNs determine that the processing of aggregation queries should be done in-network rather than by the sink collecting the raw data of each node. Some privacy-sensitive application scenarios require us to consider data privacy in addition to efficiency while dealing with aggregation queries. Data privacy in WSNs means that the data of any node can not be obtained by the nodes other than the sink.

So far, all the existing data aggregation schemes [12,13,14,15,16,17,18,19,20,21,22,23] preserving data privacy in WSNs have some apparent drawbacks. The data privacy of some of these schemes is easily attacked, some of these schemes have high communication overhead and low efficiency, and all of these schemes can not deal with min()/max() queries. In this paper, we propose a Ring-based Privacy-Preserving Aggregation Scheme (RiPPAS). RiPPAS performs aggregation through a pre-established ring structure. It uses a pseudonym mechanism for anonymous communication and uses a homomorphic encryption technique to add noise to the data that can easily be disclosed. Compared with the existing schemes, RiPPAS has three advantages: (1) It provides robust data privacy protection; (2) It is very communication-efficient; (3) It can handle all kinds of aggregation queries including min()/max() queries. Thus, the contribution of this paper comprises:

- Designing a ring structure used for data collection and aggregation.
- Proposing a ring-based privacy-preserving aggregation scheme (RiPPAS). RiPPAS provides robust privacy protection and has high efficiency. Furthermore, RiPPAS can deal with all kinds of aggregation queries.

The rest of the paper is organized as follows. Section 2 introduces the related works in this area; Section 3 gives some necessary preliminaries; Section 4 describes the ring structure used by RiPPAS; Section 5 and Section 6 gives details as to how RiPPAS handles sum() queries and min()/max() queries respectively. Section 7 gives the theoretical analysis and simulation results; Section 8 concludes the whole paper.

## 2. Related Works

Reference [24] gives a sketch of data aggregation techniques in WSNs. We focus on data privacy of WSNs in this paper. A survey of privacy preservation in WSNs is given by [19]. There are some works [12,13,14,15,16,17,18,19,20,21,22] that focus on processing aggregation queries with privacy preservation in WSNs. Reference [23] designs a middleware between the network layer and MAC layer to automatically aggregate the data from the network layer.

Reference [18] proposes two aggregation methods CPDA and SMART with privacy preservation, both of which can only deal with additive aggregation function sum(). Other aggregation queries like count(), average() and variance() can be converted to sum() queries. Both of these two methods assume that a pair-wise key model has been pre-established, i.e., each pair of neighboring nodes shares an independent key for encrypting/decrypting messages between them. Therefore, any compromised node has no effect on the pair-wise keys shared by the other pairs of nodes. CPDA is based on a cluster structure. After building clusters, each node generates some random numbers locally, then gets some values calculated by a series of network-wide shared functions which take its local raw data and the generated random numbers as arguments. One of these values remains local, and other values are distributed within the cluster. Each node adds all the received values to its local value and sends the additive result to the cluster head. After the cluster head receives the results from all cluster members, it can get the sum of the private data of all cluster members by solving a linear equation. The intra-cluster information exchange process does not reveal the raw data of any node. Next, all the cluster heads are organized as a tree rooted at the sink. The aggregation of all cluster heads’ results are performed along the tree in a bottom-up manner. SMART is a tree-based aggregation method and has three steps. For initialization, all the nodes are organized as a spanning tree rooted at the sink. In the first step, each node divides its private data into *J* pieces, stores one of these pieces locally, then sends the other J−1 pieces randomly to different *h*-hop neighbors (Normally set h=1). In the second step, each node adds all the received pieces to the locally stored piece. In the third step, in a bottom-up manner along the spanning tree, each node aggregates its local data and all the data from its children, then sends the result to its parent. Both CPDA and SMART methods need to exchange a large amount of data in the entire network to ensure data privacy, so their communication efficiency is not high. In addition, these two methods can not handle min()/max() queries.

In [12], a method named HEEPP similar to the SMART method is proposed to handle sum() queries while preserving data privacy. Unlike the SMART method, HEEPP only lets the leaf nodes in the spanning tree split their private data, while the intermediate nodes do not need to split their data. Each leaf node divides its private data into *R* pieces, and sends R−1 pieces to different *h*-hop neighbors, where *R* is a random integer in [1,K] and *K* is the maximum number of divided pieces set by users. HEEPP assumes that any two neighboring nodes share an independent key for point-to-point secure communication. Like the SMART method, HEEPP also needs to exchange a large amount of data in the network, so it has a relatively high communication overhead too. Also, HEEPP cannot handle min()/max() queries.

By improving the SMART method, Reference [17] also proposes a method named PEPDA to process sum() queries with privacy preservation. PEPDA improves the SMART methods in two aspects. In the first aspect, PEPPA splits the private data of each node into items of a power series (the former *J* terms). The power series is set to ensure that the first piece has the largest proportion in the private data, and the following pieces have gradually decreased proportion in the private data. Each node saves the first piece locally and sends the other J−1 pieces to different neighbors. If the data sent to a neighbor is lost, it will not affect the aggregation accuracy too much. In the second aspect, if a node does not have J−1 different neighbors, it performs no data division and sends its private data directly to its parent. PEPPA also assumes that any two neighboring nodes share an independent key. As a result of the need for massive data exchange in the network, PEPDA’s communication efficiency is also relatively low. PEPDA can not handle min()/max() queries either.

Similar to the CPDA method, a method named PAPF is proposed in [20,21] for handling sum() queries based on cluster structure. PAPF divides each cluster into multiple *p*-classes such that the size of each *p*-class meets some certain restrictions. Each node generates a series of random numbers and exchanges their random numbers within its *p*-class. Finally, each node constructs an *μ*-th order function named *p*-function. The construction process guarantees that the sum of the *j*-th (1≤j≤μ) order coefficient of all the *p*-functions in a *p*-class is equal to zero. Next, each node adds a noise calculated by its *p*-function to its private data, and sends the distorted data to the cluster head. The characteristics of the *p*-function ensure that the sum of all the distorted data in a *p*-class is equal to sum of all the private data. PAPF also assumes that any two neighboring nodes share an independent key, so point-to-point data transmission is secure. PAPF also has low communication efficiency, because it requires a lot of data exchange in the network. PAPF can not handle min()/max() queries either.

Based on homomorphic encryption technique, Reference [14,15] propose an approach for handling sum() queries with privacy preservation. The proposed method completes aggregation along a spanning tree rooted at the sink in a bottom-up manner. Each node $v_{i}$ holds a key $K_{i}$ shared with the sink only. Each $v_{i}$ encrypts its private data with function $c_{i}=Enc\left(m_{i},K_{i}\right)=m_{i}+K_{i}\left(modM\right)$, where $m_{i}$ is $v_{i}$’s private data and $c_{i}$ is the generated ciphertext. After receiving an intermediate node sum (modular sum) from all its children, all the received ciphertext and its locally generated ciphertext, then sends the result to its parent. Finally, the sink gets the result $c_{agg}=\sum c_{i}\left(modM\right)$, where $\sum c_{i}$ is the sum of the ciphertext of all the nodes participating in the aggregation. Next, the sink applies function $m_{agg}=Dec\left(c_{agg},k\right)=c_{agg}-k\left(modM\right)$ to decrypt the data. Where $k=\sum K_{i}$ is the sum of the keys of all the nodes participating in the aggregation. The decrypted value $m_{agg}$ is exactly equal to the sum of the private data of each node. In order to ensure the correctness of the calculation, *M* is set to a number larger than the sum of all nodes’ private data. This method does not require that the communication between any pair of nodes is encrypted with an independent key. However, to let the sink know which nodes are involved in the aggregation (so the sink can decrypt the data correctly), each aggregation value needs to be appended with the IDs of all the nodes participating in the aggregation. If there are a large number of nodes in the network, this additional information will involve a serious communication burden. The proposed method is only suitable for handling sum() queries.

Reference [22] also gives a privacy-preserving aggregation technique based on homomorphic encryption. The proposed method is both additively homomorphic and multiplicatively homomorphic, i.e., it can be used for finding the sum or the product of the data of multiple nodes. As a result of taking into account multiplicatively homomorphic situations, the encryption techniques are more complicated than the ones in [14,15], and the ciphertext sent by each node is much longer. From the perspective of data aggregation, the extra computation and communication costs are not worth it, since finding the product of data from some nodes is not a typical aggregation query and is rarely issued by users.

Reference [13] proposes a scheme to alleviate the network congestion by data aggregation. When a node’s communication bandwidth is less than the rate of generating data, the node will save the data in its buffer and upload it when the network is idle. When the data in its buffer is about to overflow, the node will aggregate all the data in its buffer and send out the aggregation value. In the process of data aggregation, the homomorphic encryption method proposed in [14,15] is used. Some additional information is added to each packet for integrity verification. This additional information makes the communication cost of the proposed method higher than that of the method in [14,15]. Furthermore, the proposed method is only suitable for handling sum() queries.

In summary, all the existing methods have two shortcomings: (1) low communication efficiency; (2) can not handle min()/max() queries.

## 3. Preliminaries

Before sensor nodes are deployed, the sink assigns each node $v_{i}$: (1) a fixed ID (also denoted by $v_{i}$); (2) an independent key $K_{i}$; (3) *m* pseudonyms $\left\{PN_{i}^{1},PN_{i}^{2},\cdots ,PN_{i}^{m}\right\}$; (4) an identical function R(K,t). Different nodes do not have any pseudonyms in common. R(k,t) is a function taking *K* and *t* as seeds to generate a pseudo random number, where *K* is the key assigned to the node and *t* is the query’s sequence number. The notations we use in this paper and their meanings can be found in Table 1. The sink uses a table to record the keys and pseudonyms assigned to each node. At regular intervals, the sink reassigns pseudonyms for all nodes. The new pseudonyms assigned to $v_{i}$ are encrypted with $K_{i}$. The table maintained by the sink is as shown in Table 2. The information of each node is recorded by a tuple in the table. After the ring structure used for data collection and aggregation has just been established, each node $v_{i}$ encrypts its location with $K_{i}$ and uploads it to the sink. The sink decrypts $v_{i}$’s location using $K_{i}$, and saves its location into the table.

We assume that all the nodes use one public channel to communicate with each other. All the links in the given network are bidirectional, i.e., if *v* can receive the signal from *u* then *u* can receive the signal from *v*. After the nodes are deployed, each pair of neighboring nodes share an independent key to encrypt/decrypt the messages between them. Refer to [25,26,27] for how to generate pairwise shared keys amongst neighboring nodes. It should be noted that when building ring structure and processing min()/max() queries, there is no need to encrypt point-to-point communications.

We assume that the goal of adversaries is to get the private data of some certain nodes. Adversaries may attack data privacy in two ways: eavesdropping on the communication channel or capturing nodes. Adversaries launches eavesdropping attack by deploying some wireless communication devices in the network area which can eavesdropping on nearby data transmissions. If data transmission is done using cryptographic encryption techniques, adversaries can not get the real content of the data only through eavesdropping. Adversaries launch a capturing attack by capturing some sensor nodes in the network physically. Once adversaries capture a node, they get the data of the node and all the keys maintained by the node for encryption/decryption, so the node becomes a compromised node and adversaries can decrypt all the packets sent to the node. Furthermore, adversaries can launch a collusion attack by gathering all the data from the eavesdropping devices and the compromised nodes. Moreover, we assume that adversaries can not approximate the sender’s location by measuring signal strength in a collusive way.

## 4. Ring Structure

In this section, we describe the ring structure we used for data aggregation.

### 4.1. Building Ring Structure

**Figure 1:**
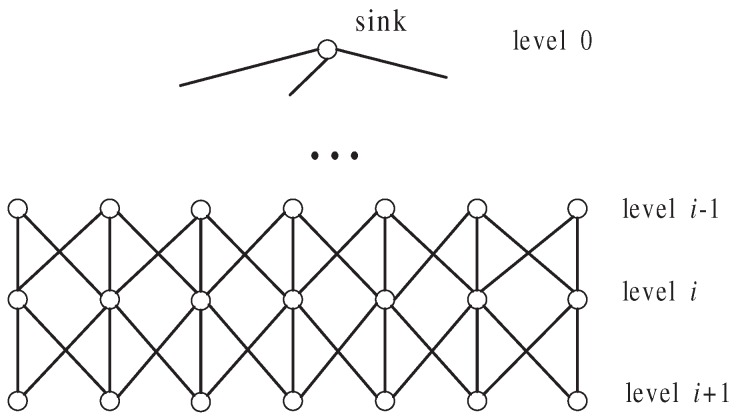
Ring Structure.

We want to build a sink-centered ring structure as shown in Figure 1, which is similar to the sink-rooted tree structure. The main difference between the ring and tree is that a node in a tree has only one parent whereas a node in a ring can have more than one predecessor (equivalent to the parent in the tree). In the ring structure, nodes are organized in levels. The level of the sink is 0 and the level of the sink’s direct neighbor is 1. The farther a node is from the sink, the higher level the node is. We use $level(v_{i})$ to denote $v_{i}$’s level. $v_{i}$ has one or more predecessors in $level(v_{i})-1$, each of which can forward the messages that $v_{i}$ sends to the sink. We use $Pred\left(v_{i}\right)=\left\{v_{j},v_{k},\cdots \right\}$ to denote the set of $v_{i}$’s predecessors. $v_{i}$ has some successors in $level(v_{i})+1$ and $v_{i}$ can forward the messages they send to the sink. $v_{i}$ uses variable $SN(v_{i})$ to record the number of its successors. A node $v_{i}$ with no successors (i.e., $SN(v_{i})=0$) is called an *outer node*. A node who has successors is called an *inner node*.

After all the nodes are deployed, the sink broadcasts a BUILD-RING message <sink,0> including its ID and level to initialize the process of building a ring. When a node $v_{i}$ receives a BUILD-RING message (let the message be $<v_{j},level\left(v_{j}\right)>)$ for the first time, $v_{i}$ sets its own level to $level\left(v_{i}\right)=level\left(v_{j}\right)+1$ and records $v_{j}$ as one of its predecessors, then broadcasts a BUILD-RING message $<v_{i},level\left(v_{i}\right)>$. While $v_{i}$ receives another BUILD-RING message $<v_{k},level\left(v_{k}\right)>$ after broadcasting BUILD-RING message, it checks $level(v_{k})$. If $level\left(v_{k}\right)=level\left(v_{i}\right)-1$, then $v_{i}$ records $v_{k}$ as one of its predecessors. If $level\left(v_{k}\right)=level\left(v_{i}\right)+1$, it means that $v_{k}$ is $v_{i}$’s successor, so $v_{i}$ adds 1 to its $SN(v_{i})$. After the ring is established, each node $v_{i}$ knows: (1) its level $level(v_{i})$; (2) the set of its predecessors $Pred(v_{i})$; (3) the number of its successors $SN(v_{i})$. The detail pseudo-codes for building the ring are given by Algorithm 1.

**Algorithm 1:** Building Ring Structure **Input**: a sensor network **Output**: a ring structure centered at the sink /*
Codes for each node $v_{i}\neq thesink$. The process starts with the sink  broadcasting a BUILD-RING message <sink,0>.          */ 
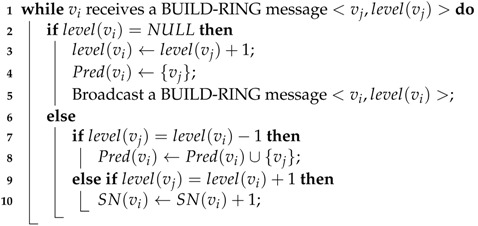


### 4.2. Data Collection/Aggregation in the Ring

The built-up ring structure can be used for data collection/aggregation of the whole network. The header of each packet used for data collection/aggregation contains the following information: (1) the receiver’s ID (which can be a broadcast address); (2) the sender’s ID; (3) the sender’s level. The header of a packet is always being transmitted in plaintext. The process of data collection/aggregation is as follows.

If node $v_{i}$ is an outer node, i.e., $SN(v_{i})=0$, then $v_{i}$ sends its data to its predecessor(s). There are two ways to send the data:

- **Real-name ciphertext unicasting**. $v_{i}$ randomly pick a predecessor $v_{j}$ from $Pred(v_{i})$, encrypts its data with the key shared by $v_{i}$ and $v_{j}$, and sends out a packet with the encrypted data in its data field. The header of the packet is set as follows: the receiver’s ID is set to $v_{j}$; the sender’s ID is set to $v_{i}$; the sender’s level is set to $level(v_{i})$. After receiving this packet, by reading the packet’s header, $v_{j}$ can know that the sender is $v_{i}$, so $v_{j}$ uses a corresponding key to decrypt the data field of the packet and gets $v_{i}$’s data.
- **Anonymous plaintext broadcasting**. $v_{i}$ constructs a packet and puts its data in the packet’s data field in plaintext, then broadcasts the packet. The header of the packet is set as follows: the receiver’s ID is set to the broadcast address; the sender’s ID is set to blank; the sender’s level is set to $level(v_{i})$. In this way, every node receiving the packet is able to obtain $v_{i}$’s data but has no way of distinguishing the packet’s sender. As stated in [28,29], we assume that the sensor nodes have ability to obfuscate address fields in their Medium Access Control (MAC) layer header, so that a compromised node can not know the sender’s identity by parsing a packet’s MAC layer header.

While node $v_{i}$ hears a packet sent from $level(v_{i})+1$, no matter the receiver is $v_{i}$ or not, $v_{i}$ adds 1 to its $hear(v_{i})$, where $hear(v_{i})$ indicates the number of the packets $v_{i}$ hears from its successors in the current round of collection/aggregation. The characteristics of radio transmission determine that the nature of all data transmission is broadcasting. Therefore, even if a packet’s destination address is not $v_{i}$, as long as $v_{i}$ is in the sender’s communication range, it can hear the packet and know the sender’s level by reading packet header. If $hear\left(v_{i}\right)=SN\left(v_{i}\right)$, it means that all the successors of $v_{i}$ have sent data in this round of data collection/aggregation, so $v_{i}$ integrates all the received data (decrypts first if it is encrypted) into a packet and sends the packet to its predecessor(s) by either real-name ciphertext unicasting or anonymous plaintext broadcasting.

In the process of collection/aggregation, the failures or compromises of nodes may cause that some intermediate nodes keep waiting for hearing from its successors. For this reason, we set a timer for each node. The duration of each node’s timer is determined by the size of the network and the node’s level. When the timer expires, no matter if a node has heard from all its successors or not, it will upload its local result anyway.

### 4.3. Advantages of Ring-Based Collection/Aggregation

Compared with tree-based collection/aggregation, ring-based collection/aggregation has an important advantage such that the data of each node goes to the sink along unfixed path. Even if adversaries can get the data in some packets, it is very difficult for them to trace the sources of the data. Here, we also stipulate that each node $v_{i}$ refuses to answer any query about a packet’s source unless $v_{i}$ receives a message from the sink encrypted with $K_{i}$.

Suppose that we collect data by real-name ciphertext unicasting. In a tree structure, if adversaries capture a node $v_{i}$, they will be able to parse the data of all the nodes in the subtree rooted at $v_{i}$. Since the subtree rooted at $v_{i}$ is fixed, adversaries can perform long-term behavior analysis for the nodes in the subtree. In a ring structure, even if adversaries capture node $v_{i}$, because $v_{i}$ receives data from an uncertain set of nodes, it is very hard for the adversaries to perform a long time analysis for some nodes.

When processing a min()/max() queries, each data is appended with a pseudonym to indicate the source of the data. Ring structure is very beneficial to the designed pseudonym mechanism. Suppose that adversaries have captured a node $v_{i}$. If we use tree structure for aggregation, since the sub-tree rooted at $v_{j}$ is fixed, by listening messages for a long time, $v_{i}$ can easily associate a node in the subtree with a set of pseudonyms, especially when there are fewer nodes in the subtree. If we use ring structure for aggregation, it is very hard for adversaries to know the corresponding relationship between pseudonyms and the nodes.

As with the tree structure, the ring structure does not require synchronization in the network. The process of data collection/aggregation is entirely message-driven.

## 5. Handling sum() Queries

In this section, we describe how RiPPAS handles sum() queries. Some other types of queries like count(), average() and variance() can be converted to sum() queries [14,15]. While performing aggregation in the ring structure, RiPPAS uses homomorphic encryption technique to add noise to outer nodes’ private data. Let $d_{i}$ denote the private data generated by node $v_{i}$ in this round of aggregation. The procedure for RiPPAS handling sum() queries is given by Algorithm 2.

**Algorithm 2:** Dealing with sum() queries **Input**: a sensor network with ring structure established **Output**: the sum of all nodes’ data 
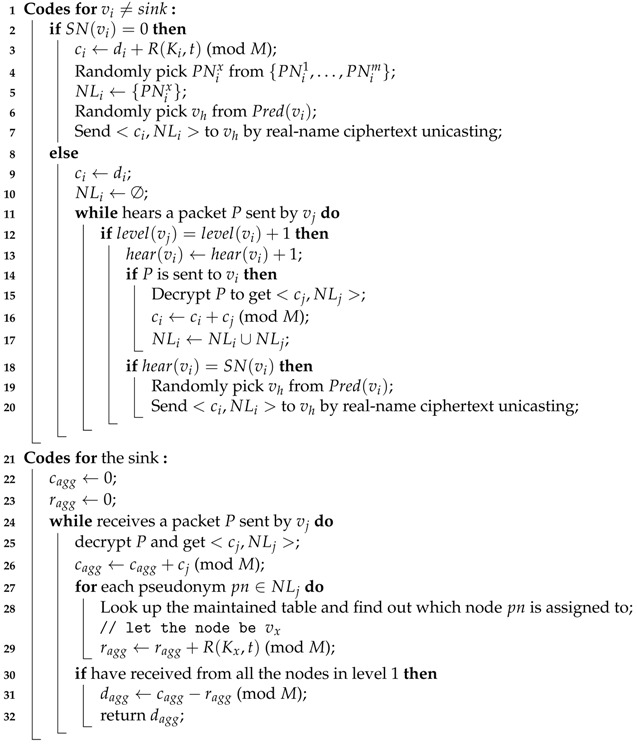


For an outer node $v_{i}$: it adds noise $R(K_{i},t)$ to its the private data $d_{i}$ and generates noisy data

$$c_{i}=d_{i}+R\left(K_{i},t\right)\;(\mathrm{mod}\;M)$$

where *R* is a function used by nodes to generate a pseudo random value, *t* is the sequence number of the current query, and *M* is a predetermined constant satisfying $M\geq \sum_{\mathrm{for}\;\mathrm{all}\;v_{i}}d_{i}$. Next, $v_{i}$ randomly picks a pseudonym from its pseudonym set $\left\{PN_{i}^{1},PN_{i}^{2},\cdots ,PN_{i}^{m}\right\}$ and adds it to the set $NL_{i}$ (Line 4–5 in Algorithm 2). $NL_{i}$ records which nodes have added noise to the result $c_{i}$ in form of pseudonym. Finally, $v_{i}$ sends the data $<c_{i},NL_{i}>$ to a randomly selected successor $v_{h}$ by real-name ciphertext unicasting (Line 6–7 in Algorithm 2).

For an inner node $v_{i}$: if $v_{i}$ hears a packet *P* sent from its next level (confirmed by checking the sender’s level in *P*’s header), it adds 1 to its local variable $hear(v_{i})$ (Line 12–13 in Algorithm 2). If *P* is sent to $v_{i}$ (confirmed by checking the receiver’s ID in *P*’s header), according to the sender’s ID (let the sender be $v_{j}$), $v_{i}$ finds the corresponding key to decrypt *P* and gets its data $<c_{j},NL_{j}>$ (Line 14–15 in Algorithm 2). Let $<c_{j_{1}},NL_{j_{1}}>,\cdots ,<c_{j_{k}},NL_{j_{k}}>$ be all the data received by $v_{i}$. Then $v_{i}$ sets

$$c_{i}=d_{i}+\sum_{x=1}^{k}c_{j_{x}}\;(\mathrm{mod}\;M)$$

and

$$NL_{i}=\overset{k}{\underset{x=1}{⋃}}NL_{j_{x}}$$

If all its successors have uploaded their data, i.e., $hear\left(v_{i}\right)=SN\left(v_{i}\right)$, $v_{i}$ sends $<c_{i},NL_{i}>$ to a randomly selected predecessor $v_{h}$ by real-name ciphertext unicasting (Line 19–20 in Algorithm 2).

The sink does the following: decrypt each received packet to get its data $<c_{j},NL_{j}>$, and use variable $c_{agg}$ to aggregate all the received $c_{j}$, i.e.,

$$c_{agg}=\sum_{i=1}^{n}c_{i}\;(\mathrm{mod}\;M)$$

For each pseudonym pn in each received $NL_{j}$, look up the maintained table and find out which node pn is assigned to (let the node be $v_{x}$). Calculate the noise $R(K_{x},t)$ that $v_{x}$ adds to the result. Use variable $r_{agg}$ to record the sum of all the added noise (Line 28–29 in Algorithm 2), i.e.,

$$r_{agg}=\sum_{\mathrm{for}\;\mathrm{each}\;\mathrm{outer}\;\mathrm{node}\;v_{i}}R(K_{i},t)\;(\mathrm{mod}\;M)$$

If has received from all the nodes in the first level, de-noise the result $c_{agg}$ by the function

$$d_{agg}=c_{agg}-r_{agg}\;(\mathrm{mod}\;M)$$

Return the result $d_{agg}$ to users(Line 31–32 in Algorithm 2). $d_{agg}$ is the sum of each node’s private data.

In the whole aggregation process, the local aggregation result $c_{i}$ uploaded by each node $v_{i}$ has constant length. Only outer nodes need to add noise to their results and append their pseudonyms. All inner nodes do not need to add noise or append pseudonyms. Therefore, the communication efficiency for RiPPAS handling sum() queries is very high.

## 6. Handling min()/max() Queries

In this section, we describe how RiPPAS handles min()/max() queries. By issuing a min()/max() query, users are concerned with the minimum/maximum value of the data in the entire network, as well as the node that generates the value and its location. When processing a min()/max() query, RiPPAS focuses on protection of the source of the minimum/maximum value. It uses a pseudonym mechanism for anonymous communication. The process for RiPPAS handling max() queries is given by Algorithm 3.

**Algorithm 3:** Dealing with max() query **Input**: a sensor network with ring structure established **Output**: the maximum value of all nodes’ data, the node generating the maximum value and its location 
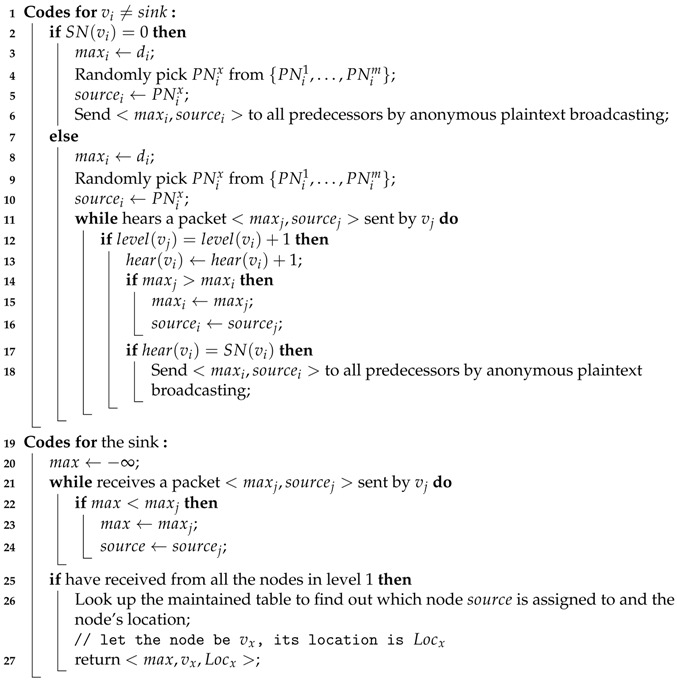


The nodes in the ring upload their local results level by level. Data packets are transmitted by anonymous plaintext broadcasting, so the receiver of a packet has no information about the sender except for its level in the ring. The data packet uploaded by node $v_{i}$ has the form $<max_{i},source_{i}>$, where $max_{i}$ is $v_{i}$’s local maximum value (the maximum value of the data received by $v_{i}$ and its private data), $source_{i}$ is the pseudonym of the node who generate $max_{i}$. $source_{i}$ is uploaded so that the sink can know the source of the maximum value by looking up the pseudonym table.

First, each outer node sends its data with a randomly picked pseudonym to all its predecessors by anonymous plaintext broadcasting (Lines 3–6 in Algorithm 3). After an inner node receives data from all its successors, it gets the maximum value from the set including all the received data and its own data, attaches the corresponding pseudonym (if the maximum value is $v_{i}$’s own data, $v_{i}$ randomly picks a pseudonym and appends it to the data), then sends the maximum value to all its predecessors by anonymous plaintext broadcasting (Lines 8–18 in Algorithm 3). Finally, the sink gets the maximum value of the entire network and its source’s pseudonym. By looking up the maintained table, the sink will know the source’s ID and location (Lines 21–27 in Algorithm 3).

When processing a min()/max() query, RiPPAS guarantees that as long as adversaries do not capture the source node of the maximum/minimum value, it is impossible for them to know the source node’s ID and its location. The shortcoming of RiPPAS is that adversaries can get the maximum/minimum value by eavesdropping even if they do not capture any nodes. However, As we have stated before, the most import information of min()/max() aggregation is not the maximum/minimum value itself, but the node who generates the maximum/minimum value and its location. Thus, to process min()/max() queries, each node upload its data by anonymous plaintext broadcasting. If users want to prevent the disclosure of the maximum/minimum value information, we can modify RiPPAS and let each node upload its data by real-name ciphertext unicasting.

## 7. Evaluation

The performance of RiPPAS is evaluated in this section. The evaluation is done in three aspects: (a) privacy preservation; (b) communication overhead; (c) aggregation accuracy.

For sum() queries, we compare RiPPAS with three other aggregation methods: (1) the SMART method proposed in [18]; (2) the HEEPP method proposed in [12]; (3) the method based on homomorphic encryption proposed in [14,15] (denoted by “HOMOENC”). All the other three methods can not deal with min()/max() queries.

For min()/max() queries, we compare three aggregation schemes: (1) the RiPPAS scheme that each node uploads data by anonymous plaintext broadcasting (denoted by RiPPAS_APB); (2) the RiPPAS scheme that each node uploads data by real-name ciphertext unicasting (denoted by RiPPAS_RCU); (3) the tree-based aggregation scheme proposed in [30] (denoted by EADAT) with the improvement that each pair of neighbors use an independent key to encrypt/decrypt the communication between them. In EADAT, all the nodes are organized as a spanning tree rooted at the sink. In a bottom-up manner, each node sends its local maximum/minimum value with the ID of the data’s source to its parent. Note that EADAT deals with min()/max() queries without any data privacy concern. Since all the existing methods can not deal with min()/max() queries with privacy preservation, we choose the most typical method to compare with the proposed methods.

We use a simulator written in C++ to do the evaluation. The simulation environment is set as follows: 2500 sensor nodes are randomly deployed in a 1500 m × 1500 m area. Each node is assigned m=20 pseudonyms. As a result, the sink uses a 120 M table to records the information of all the sensor nodes. The effective transmitting range of each sensor node is set to 50 m, so each node has 8.7 neighbors on average. We divide the time into equal-sized time slots. The transmission of any data packet can be done in one time slot. Each node can only send or receive one data packet (can not both send and receive) in one time slot. Each data packet has a 7-byte header containing the information of packet type (1 byte), receiver’s ID (2 bytes), sender’s ID (2 bytes), sender’s level in the ring (1 byte) and packet length (1 byte). The data field of each packet contains up to 50 bytes. We synthesize the sampled data for each node according to the data set in [31]. Each node samples four kinds of data: temperature, humidity, light, voltage. To get each point in the result figures, we conduct 10 simulations and take their average value. When pair-wise encrypted communication is required in SMART, HEEPP, RiPPAS_RCU and EADAT, we use the encryption/decryption technique in [32].

### 7.1. Privacy Preservation

Next, for an arbitrary node *v* in the given network, we will analyse the probability that *v*’s private data is disclosed to adversaries in different aggregation schemes. Here, we assume that *v* itself is not captured by adversaries. As we stated in Section 3, adversaries can launch collusion attack by gathering all the data from the eavesdropping devices and the captured nodes. As a result, for each wireless link in the given network, adversaries has a certain probability to break the link. “Break a link” means that adversaries know the key used for encryption/decryption, so they can get all the data transmitted on the link. We use $q_{b}$ to denote the probability that any link is broken by adversaries.

#### 7.1.1. Privacy Preservation for sum() Queries

Let us analyse the privacy preservation for RiPPAS processing sum() queries. For an outer node *v*, since it adds noise to its private data and only the sink has the key to de-noise the data, adversaries can not get *v*’s private data if they do not capture *v*. Let $P_{v}\left(q_{b}\right)$ be the probability that *v*’s data is disclosed under the setting of $q_{b}$. For outer node *v* in RiPPAS, we have

(1) $$P_{v}\left(q_{b}\right)=0$$

For an inner node *v*, let PreSuc(v) be the set including *v*’s successors who upload their aggregation reults to *v* and *v*’s predecessor to whom *v* upload its result, i.e.,

(2) PreSuc(v)={u|uuploadsitsresultstov}∪{u|vuploadsitsresulttou}

Adversaries can only get the *v*’s data by breaking all the links connecting *v* and the nodes in PreSuc(v). Therefore, for inner node *v* in RiPPAS, we have

(3) $$P_{v}\left(q_{b}\right)=\sum_{k=1}^{max\_d}Pr\left\{|PreSuc\left(v\right)|=k\right\}\cdot q_{b}^{k}$$

where Pr{|PreSuc(v)|=k} is the probability that |PreSuc(v)|=k and max_d is the maximum degree in the network.

For sum() queries, SMART organizes all the node as a spanning tree. Each node divides its private data into *J* pieces, then sends J−1 pieces to different neighbors. Define node set InOut(v) as

(4) InOut(v)={u|usendsitspiecetov}∪{u|vsendsitspiecetou}

In SMART, adversaries can only get *v*’s data by breaking all the links connecting *v* and the nodes in InOut(v). Therefore, for any node *v* in SMART, we have

(5) $$P_{v}\left(q_{b}\right)=\sum_{k=1}^{max\_d}Pr\left\{|InOut\left(v\right)|=k\right\}\cdot q_{b}^{k}$$

HEEPP is similar to SMART. In HEEPP, only leaf nodes in the spanning tree split their private data. Each leaf node divides its private data into *R* pieces, and sends the R−1 pieces to different neighbors, where *R* is a random integer in [1,K]. Similar to SMART, for a leaf node in HEEPP, the probability that adversaries can get *v*’s private data is given by Equation (5). In HEEPP, adversaries can get an intermediate node’s private data by getting all the data its children send to the node and the data the node send to its parent. Define node set PaCh(v) as

(6) PaCh(v)={u|uisv’schildoruisv’sparent}

Therefore, for intermediate node *v* in HEEPP, we have

(7) $$P_{v}\left(q_{b}\right)=\sum_{k=1}^{max\_d}Pr\left\{|PaCh\left(v\right)|=k\right\}\cdot q_{b}^{k}$$

HOMOENC uses homomorphic encryption to process sum() queries. Each node encrypts its private data and only the sink can decrypt the data. All the aggregation is done on ciphertext. Adversaries can not get a node’s private data anyway (if they do not capture the node itself). Thus, for any node *v* in HOMOENC, we have

$$P_{v}\left(q_{b}\right)=0$$

Both RiPPAS and HOMOENC use homomorphic encryption for secure data aggregation. In HOMOENC, every node encrypts its local result before uploading it. The additively homomorphic encryption function used by HOMOENC can be seen as adding some noise to the real data. The noise is generated by a pseudo random function taking node’s key as seed. For a node who add noise to its private data, only the sink can de-noise the noisy data, so adversaries have no way to get the node’s private data (except for capturing the node itself). From the perspective of data privacy, letting every node use additively homomorphic encryption function to encrypt its local result is very safe. However, to let the sink de-noise the result correctly, each node adding noise to the result has to append its ID to the result, which poses a heavy burden on communication.

The encryption technique used in RiPPAS is almost as same as the one used in HOMOENC. The only difference is that in RiPPAS, the function used by each node to generate a pseudo random noise takes both the node’s key and the query’s sequence number as seeds, which makes the homomorphic encryption in RiPPAS is more secure. In the process of aggregation, the outer nodes in the ring have to upload their private data, so their data privacy is vulnerable to attack. On the contrary, the data uploaded by an inner node is the aggregation result of its private data and the data from its successors, so it is very difficult for adversaries to attack their data privacy. In RiPPAS, we only let the outer nodes encrypt their data. For an outer node, adversaries have no way to get its private data. For an inner node, the only way adversaries can get its private data is by getting the data the node sends out and all the data sent into the node, which is still a very hard task for adversaries. Compared to HOMOENC, RiPPAS is a little more vulnerable. However, only the outer nodes in RiPPAS have to append its ID to the aggregation result, which makes RiPPAS much more communication efficient than HOMOENC.

**Figure 2:**
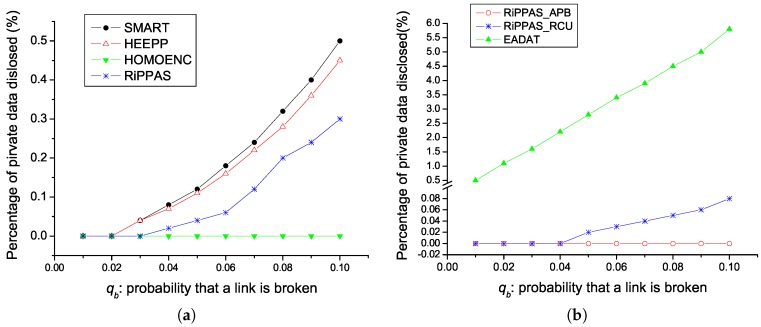
Privacy preservation performance. (**a**) Handling sum() queries on temperature; (**b**) Handling min()/max() queries on temperature.

In our simulation we set J=3 for SMART and set K=5 for HEEPP. The effectiveness of privacy preservation is measured by the percentage of the nodes whose data is disclosed to adversaries. The comparison results of privacy preservation performance for processing sum() queries on temperature is given by Figure 2a. Except for HOMOENC, the larger $q_{b}$, the weaker privacy preservation. HOMOENC always gives the best performance. Since HOMOENC uses homomorphic encryption technique, it is impossible for adversaries to obtain a node’s private data. RiPPAS outperforms SMART and HEEPP in this aspect. In RiPPAS, outer nodes’ data can not be disclosed anyway. Adversaries cannot get an inner node’s data unless they get all the data the node receives and the data the node sends out. In SMART and HEEPP, it is relatively easier for adversaries to get a node’s private data.

By ring-based aggregation, RiPPAS has more advantages in addition to the simulation results shown. Suppose that adversaries want to make sure to get a certain node *v*’s private data. For an outer node *v*, if *v* is not captured, adversaries can not get *v*’s private data anyway. Recall that each node uploads its aggregation result to a randomly picked predecessor. Adversaries will never know which nodes upload their results to *v* and to which node *v* uploads its result. Therefore, for an inner node *v*, if *v* is not captured, to make sure to get *v*’s private data, adversaries have to break all the links connecting *v* with all its predecessors and successors. Apparently, it is a very hard task for adversaries. Compared with a tree-based method, the ring-based aggregation scheme makes it harder for adversaries to attack data privacy. Furthermore, as we state in Section 4.3, ring-based aggregation can effectively prevent attackers from performing long-term behavior analysis on some nodes.

#### 7.1.2. Privacy Preservation for min()/max() Queries

For a min()/max() query, it is not very meaningful to know the maximum/minimum value in the whole network without knowing its source. For such kind of queries, the disclosure of node *v*’s data privacy is defined as adversaries can get *v*’s private data and know that *v* is the source of the data. In the following, we only discuss the case of processing a max() query. The case of processing a min() query is similar.

In the RiPPAS_APB scheme, nodes upload their data by anonymous plaintext broadcasting. By eavesdropping, adversaries can easily get the data in a packet by eavesdropping, but they cannot know who the sender of the packet is. On the other hand, during the aggregation process, each node uploads its local maximum value with the pseudonym of the value’s source and only the sink knows which node the pseudonym corresponds to. By eavesdropping on the transmission of a packet, adversaries cannot determine the source of the maximum value in the packet, i.e., adversaries can not relate any data to its real source. Thus, in RiPPAS_APB, the probability that any node *v*’s data privacy is disclosed is given by

$$P_{v}\left(q_{b}\right)=0$$

In the RiPPAS_RCU scheme, nodes upload their data by real-name ciphertext unicasting. Ciphertext transmission makes it relatively difficult for adversaries to break a link. However, once adversaries beak a link and get a packet transmitted on the link, they can know the real ID of the packet’s sender, which gives them opportunities to attack a node’s data privacy. If *v*’s private data is its local maximum value, *v* will append a randomly selected pseudonym to its private data and send its real-name. In this case, if adversaries can get all the packets *v* receives and the packet *v* sends out, they can distinguish that the pseudonym in the packet *v* sends out is different from all the pseudonyms in the packets *v* receives, and know that the data *v* sends out is generated by *v* itself. Therefore, if *v*’s private data is its local maximum value, adversaries can get *v*’s private data and know that *v* is the source of the data by breaking all the links connecting *v* and the nodes in PreSuc(v). Here, PreSuc(v) is the node set defined by Equation (2). For any node in the RiPPAS_RCU scheme, we have

(8) $$P_{v}\left(q_{b}\right)=P_{v}^{max}\cdot \sum_{k=1}^{max\_d}Pr\left\{|PreSuc\left(v\right)|=k\right\}\cdot q_{b}^{k}$$

where $P_{v}^{max}$ is the probability that *v*’s private data is its local maximum value.

In EADAT, each node in the spanning tree sends its local maximum value and the ID of the value’s source to its parent. Let *u* be one of *v*’s ancestors in the tree. Adversaries can get node *v*’s private data by breaking the link between *u* and *u*’s parent in the context that *v*’s private data is the local maximum value of *u*. Therefore, for any node *v* in EADAT, we have

(9) $$P_{v}\left(q_{b}\right)=\sum_{u\in AN(v)}P_{v,u}^{max}\cdot q_{b}$$

where AN(v) is the set of *v*’s ancestors in the tree and $P_{v,u}^{max}$ is the probability that *v*’s private data is the local maximum value of *u*.

The privacy preservation performance for processing min()/max() queries on temperature is given by Figure 2b. We can see that, except for RiPPAS_APB, the larger $q_{b}$ is, the weaker the privacy preservation is. In RiPPAS_APB, it is impossible for adversaries to get any node’s private data unless they capture the node itself, so RiPPAS_APB outperforms RiPPAS_RCU. Since EADAT does not concern the data privacy, it is much worse than the proposed two schemes in the aspect of privacy preservation.

### 7.2. Communication Overhead

We compare the communication overheads for different schemes processing aggregation queries on different attributes. Recall that SMART, HEEPP, HOMOENC and EADAT all require a pre-established tree structure, and RiPPAS requires to build a ring structure before aggregation. We also compare the communication overheads for different schemes building the pre-established structures. Communication overhead is represented by average number of bytes sent and received by each node.

**Figure 3:**
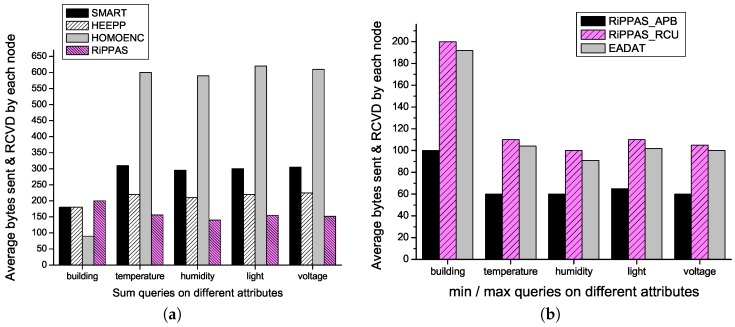
Communication overhead for processing aggregation queries on different attributes. (**a**) For sum() queries; (**b**) For min()/max() queries.

The communication overheads for SMART, HEEPP, HOMOENC and RiPPAS to process sum() queries on different attributes are given by Figure 3a, in which “building” denotes the communication overheads for building the pre-established structures. We can see that HOMOENC has the lowest building communication overhead. This is because HOMOENC does not require pair-wise key exchanging for secure point-to-point communication. The building communication overhead of RiPPAS is a little higher than the one of SMART and HEEPP, because the ring structure is a little more complex than the tree structure. The communication overheads for each scheme to process queries on different attributes are almost the same. RiPPAS always gives the best performance and is apparently better than the other three methods in this aspect. RiPPAS gains its communication efficiency because only the outer nodes add noise to their data and each node only uploads its local result once. In SMART and HEEPP, nodes split their data into pieces and exchanging data pieces among neighbors, which introduces a considerable communication overhead. HOMOENC has the worst performance. In HOMOENC, each aggregation data is uploaded with the IDs of all the nodes that participate in the aggregation. In a large-scale network, the ID list sent by a node near the sink will be extremely long.

The communication overheads for RiPPAS_APB, RiPPAS_RCU and EADAT to process min()/max() queries on different attributes are given by Figure 3b. The building communication overhead of RiPPAS_APB is much lower than the ones of RiPPAS_RCU and EADAT. Since RiPPAS_APB does not use point-to-point encrypted transmission, there is no pair-wise key exchanging phase in RiPPAS_APB. Compared with RiPPAS_RCU and EADAT, RiPPAS_APB has apparently lower communication overhead for processing min()/max() queries on different attributes. Since RiPPAS_APB does not require encrypted transmission of data packets, the packets in RiPPAS_APB are much shorter. EADAT outperforms RiPPAS_APB a little bit because there are fewer receiving activities using a tree structure to perform aggregation compared with using a ring structure.

### 7.3. Aggregation Accuracy

In ideal situations when there is no data loss in the network, all the compared schemes give 100% accurate results. However, in reality, data loss is inevitable due to the node dying, wireless interference, signal collision and processing delay. We need to measure how an aggregation scheme is affected by data loss. The comparison is conducted by aggregating on different attributes.

**Figure 4:**
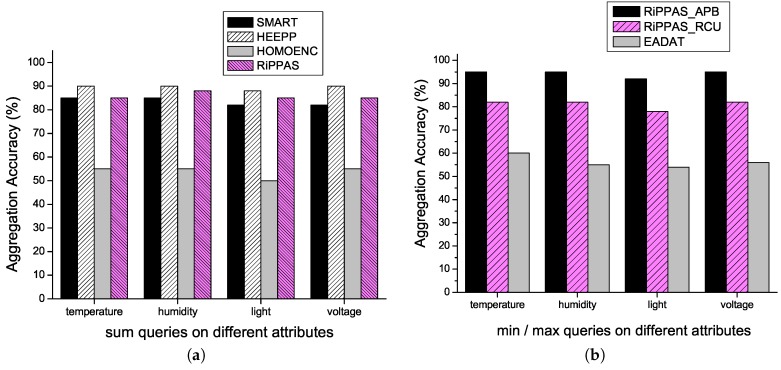
Aggregation accuracy for processing aggregation queries on different attributes. (**a**) For sum() queries; (**b**) For min()/max() queries.

The aggregation accuracy for SMART, HEEPP, HOMOENC and RiPPAS to process sum() queries on different attributes is given by Figure 4a. Each scheme has almost the same performance on different attributes. HEEPP gives the best performance in this aspect. RiPPAS gives almost the same performance with negligible gap. SMART is a little wore than HEEPP and RiPPAS, but the gap is not apparent. HOMOENC is much worse than the other three schemes. In HOMOENC, since each aggregation data is uploaded with the IDs of all the nodes who participate in the aggregation, there are many very long packets in the area near the sink. These packets are very easily to lose due to their length. Once a packet near the sink area is lost, we lose the data of a large part of the network.

The aggregation accuracy for RiPPAS_APB, RiPPAS_RCU and EADAT to process min()/max() queries on different attributes is given by Figure 4b. RiPPAS_APB gives the best performance in this aspect. Since each node uploads its local result by broadcasting in RiPPAS_APB, the maximum/minimum value can reach the sink along different paths, and data loss at a node or on a link will not affect the final result with great probability. EADAT gives the worst performance. By using tree structure for aggregation in EADAT, once a node fails to upload its data to its parent, we lose the result derived from all the data in the subtree rooted at the node.

Some detailed simulation results can be found in Table 3 and Table 4, where privacy preservation is measured by the percentage of the nodes whose data is disclosed and communication overhead is measured by the average number of bytes sent and received by each node.

## 8. Conclusions

We propose a scheme RiPPAS for aggregation with privacy preservation in wireless sensor networks. RiPPAS adopts a ring structure to perform aggregation. It uses a pseudonym mechanism for anonymous communication and uses a homomorphic encryption technique to easily add noise to the data to be disclosed. Theoretical analysis and simulation results prove that while processing sum() queries, RiPPAS has advantages in the aspects of privacy preservation and communication efficiency compared to the existing privacy-preserving aggregation methods. Furthermore, RiPPAS can process min()/max() queries whereas all the existing methods cannot. Future research directions in this area may be: providing privacy protection for more complex queries or providing differential privacy protection in distributed environments.

## Figures and Tables

**Table 1:** Notations table.

Notation	Explanation
*n*	number of sensor nodes
$v_{i}$	the *i*-th sensor node, also denotes its ID
$K_{i}$	key shared by $v_{i}$ and the sink
$PN_{i}^{1},PN_{i}^{2},\cdots ,PN_{i}^{m}$	*m* pseudonyms the sink assign to $v_{i}$
R(K,t)	function each node use to generate pseudo random number
t	sequence number of query
$level(v_{i})$	$v_{i}$’s level in the ring
$Pred\left(v_{i}\right)=\left\{v_{j},v_{k},\cdots \right\}$	set of $v_{i}$’s predecessors in the ring
$SN(v_{i})$	number of $v_{i}$’s successors in the ring
$hear(v_{i})$	number of packets $v_{i}$ hears from its successors
$d_{i}$	private data of $v_{i}$
*M*	a constant satisfying $M\geq \sum_{i=1}^{n}d_{i}$
$c_{i}$	noisy data integrated by $v_{i}$
$NL_{i}$	set of the pseudonyms of the nodes who add noise into $c_{i}$
$max_{i}$	$v_{i}$’s local maximum value
$source_{i}$	source of $max_{i}$ in form of pseudonym
$q_{b}$	probability that a link is broken
$P_{v}\left(q_{b}\right)$	probability that node *v*’s data is disclosed under the setting of $q_{b}$

**Table 2:** Table maintained by the sink.

Node ID	Location	Key	Pseudonym 1		...		Pseudonym *m*

**Table 3:** Some detailed simulation results for handling sum() queries.

Performance	Algorithms			
	SMART	HEEPP	HOMOENC	RiPPAS
Privacy preservation (%) for $q_{b}=0.01$	0	0	0	0
Privacy preservation (%) for $q_{b}=0.05$	0.12	0.11	0	0.04
Privacy preservation (%) for $q_{b}=0.1$	0.5	0.45	0	0.3
Communication overhead for sum() on temperature	305	222	594	156
Aggregation accuracy (%) for sum() on temperature	84	90	55	84

**Table 4:** Some detailed simulation results for handling min()/max() queries.

Performance	Algorithms		
	RiPPAS_APB	RiPPAS_RCU	EADAT
Privacy preservation (%) for $q_{b}=0.01$	0	0	0.5
Privacy preservation (%) for $q_{b}=0.05$	0	0.02	2.8
Privacy preservation (%) for $q_{b}=0.1$	0	0.08	5.8
Communication overhead for max() on temperature	62	109	105
Aggregation accuracy (%) for sum() on temperature	95	82	61
